# Unambiguous identification of fungi: where do we stand and how accurate and precise is fungal DNA barcoding?

**DOI:** 10.1186/s43008-020-00033-z

**Published:** 2020-07-10

**Authors:** Robert Lücking, M. Catherine Aime, Barbara Robbertse, Andrew N. Miller, Hiran A. Ariyawansa, Takayuki Aoki, Gianluigi Cardinali, Pedro W. Crous, Irina S. Druzhinina, David M. Geiser, David L. Hawksworth, Kevin D. Hyde, Laszlo Irinyi, Rajesh Jeewon, Peter R. Johnston, Paul M. Kirk, Elaine Malosso, Tom W. May, Wieland Meyer, Maarja Öpik, Vincent Robert, Marc Stadler, Marco Thines, Duong Vu, Andrey M. Yurkov, Ning Zhang, Conrad L. Schoch

**Affiliations:** 1 Botanischer Garten und Botanisches Museum, Freie Universität Berlin, Königin-Luise-Straße 6–8, 14195 Berlin, Germany; 2 International Commission on the Taxonomy of Fungi, Champaign, IL USA; 3 Department of Botany and Plant Pathology, Purdue University, West Lafayette, IN 47907 USA; 4 National Center for Biotechnology Information, National Library of Medicine, National Institutes of Health, 45 Center Drive, Bethesda, MD 20892 USA; 5 Illinois Natural History Survey, University of Illinois, 1816 South Oak Street, Champaign, IL 61820-6970 USA; 6 Department of Plant Pathology and Microbiology, College of Bio-Resources and Agriculture, National Taiwan University, Taipe City, Taiwan; 7 National Agriculture and Food Research Organization, Genetic Resources Center, 2-1-2 Kannondai, Tsukuba, Ibaraki, 305-8602 Japan; 8 Department Pharmaceutical Sciences, University of Perugia, Via Borgo 20 Giugno, 74, Perugia, Italy; 9 Westerdijk Fungal Biodiversity Institute, Uppsalalaan 8, 3584 CT Utrecht, The Netherlands; 10 Wageningen University and Research Centre (WUR), Laboratory of Phytopathology, Droevendaalsesteeg 1, 6708 PB Wageningen, The Netherlands; 11 Microbiology and Applied Genomics Group, Research Area Biochemical Technology, Institute of Chemical, Environmental & Bioscience Engineering (ICEBE), TU Wien, Vienna, Austria; 12 Jiangsu Provincial Key Lab of Organic Solid Waste Utilization, Nanjing Agricultural University, Nanjing, China; 13 Department of Plant Pathology & Environmental Microbiology, The Pennsylvania State University, University Park, PA 16802 USA; 14 Department of Life Sciences, The Natural History Museum, Cromwell Road, London, SW7 5BD UK; 15 Comparative Plant and Fungal Biology, Royal Botanic Gardens, Kew, Surrey, TW9 3DS UK; 16 Geography and Environment, University of Southampton, Southampton, SO17 1BJ UK; 17 Jilin Agricultural University, Changchun, 130118 Jilin Province China; 18 Key Laboratory for Plant Diversity and Biogeography of East Asia, Kunming Institute of Botany, Chinese Academy of Science, Kunming, 650201 Yunnan China; 19 Center of Excellence in Fungal Research, Mae Fah Luang University, Chiang Rai, 57100 Thailand; 20 World Agroforestry Centre, East and Central Asia, Kunming, 650201 Yunnan China; 21 Mushroom Research Foundation, 128 M.3 Ban Pa Deng T. Pa Pae, A. Mae Taeng, Chiang Rai, 50150 Thailand; 22 Molecular Mycology Research Laboratory, Centre for Infectious Diseases and Microbiology, Faculty of Medicine and Health, Sydney Medical School, Westmead Clinical School, Marie Bashir Institute for Infectious Diseases and Biosecurity, The University of Sydney, Westmead Hospital (Research and Education Network), Westmead Institute for Medical Research, Sydney, NSW Australia; 23 Department of Health Sciences, Faculty of Science, University of Mauritius, Reduit, Mauritius; 24 Manaaki Whenua – Landcare Research, Private Bag 92170, Auckland, 1142 New Zealand; 25 Royal Botanic Gardens, Kew, Surrey, TW9 3DS UK; 26 Universidade Federal de Pernambuco, Centro de Biociências, Departamento de Micologia, Laboratório de Hifomicetos de Folhedo, Avenida da Engenharia, s/n Cidade Universitária, Recife, PE 50.740-600 Brazil; 27 Royal Botanic Gardens Victoria, Birdwood Avenue, Melbourne, Victoria 3004 Australia; 28 University of Tartu, 40 Lai Street, 51 005 Tartu, Estonia; 29 Department Microbial Drugs, Helmholtz Centre for Infection Research, and German Centre for Infection Research (DZIF), partner site Hannover-Braunschweig, Inhoffenstrasse 7, 38124 Braunschweig, Germany; 30 Institute of Ecology, Evolution and Diversity, Goethe University, Max-von-Laue-Straße 9, 60439 Frankfurt (Main); Senckenberg Biodiversity and Climate Research Centre, Senckenberganlage 25, 60325 Frankfurt (Main), Germany; 31 Leibniz Institute DSMZ-German Collection of Microorganisms and Cell Cultures, Braunschweig, Germany; 32 Department of Plant Biology, Rutgers University, New Brunswick, NJ 08901 USA

**Keywords:** *COX1*, *COX2*, Oxford Nanopore technologies, PacBio, *RPB2*, Read placement, Species concepts, *TEF1*

## Abstract

True fungi (*Fungi*) and fungus-like organisms (e.g. *Mycetozoa*, *Oomycota*) constitute the second largest group of organisms based on global richness estimates, with around 3 million predicted species. Compared to plants and animals, fungi have simple body plans with often morphologically and ecologically obscure structures. This poses challenges for accurate and precise identifications. Here we provide a conceptual framework for the identification of fungi, encouraging the approach of integrative (polyphasic) taxonomy for species delimitation, i.e. the combination of genealogy (phylogeny), phenotype (including autecology), and reproductive biology (when feasible). This allows objective evaluation of diagnostic characters, either phenotypic or molecular or both. Verification of identifications is crucial but often neglected. Because of clade-specific evolutionary histories, there is currently no single tool for the identification of fungi, although DNA barcoding using the internal transcribed spacer (ITS) remains a first diagnosis, particularly in metabarcoding studies. Secondary DNA barcodes are increasingly implemented for groups where ITS does not provide sufficient precision. Issues of pairwise sequence similarity-based identifications and OTU clustering are discussed, and multiple sequence alignment-based phylogenetic approaches with subsequent verification are recommended as more accurate alternatives. In metabarcoding approaches, the trade-off between speed and accuracy and precision of molecular identifications must be carefully considered. Intragenomic variation of the ITS and other barcoding markers should be properly documented, as phylotype diversity is not necessarily a proxy of species richness. Important strategies to improve molecular identification of fungi are: (1) broadly document intraspecific and intragenomic variation of barcoding markers; (2) substantially expand sequence repositories, focusing on undersampled clades and missing taxa; (3) improve curation of sequence labels in primary repositories and substantially increase the number of sequences based on verified material; (4) link sequence data to digital information of voucher specimens including imagery. In parallel, technological improvements to genome sequencing offer promising alternatives to DNA barcoding in the future. Despite the prevalence of DNA-based fungal taxonomy, phenotype-based approaches remain an important strategy to catalog the global diversity of fungi and establish initial species hypotheses.

## INTRODUCTION

**Fig. 1:**
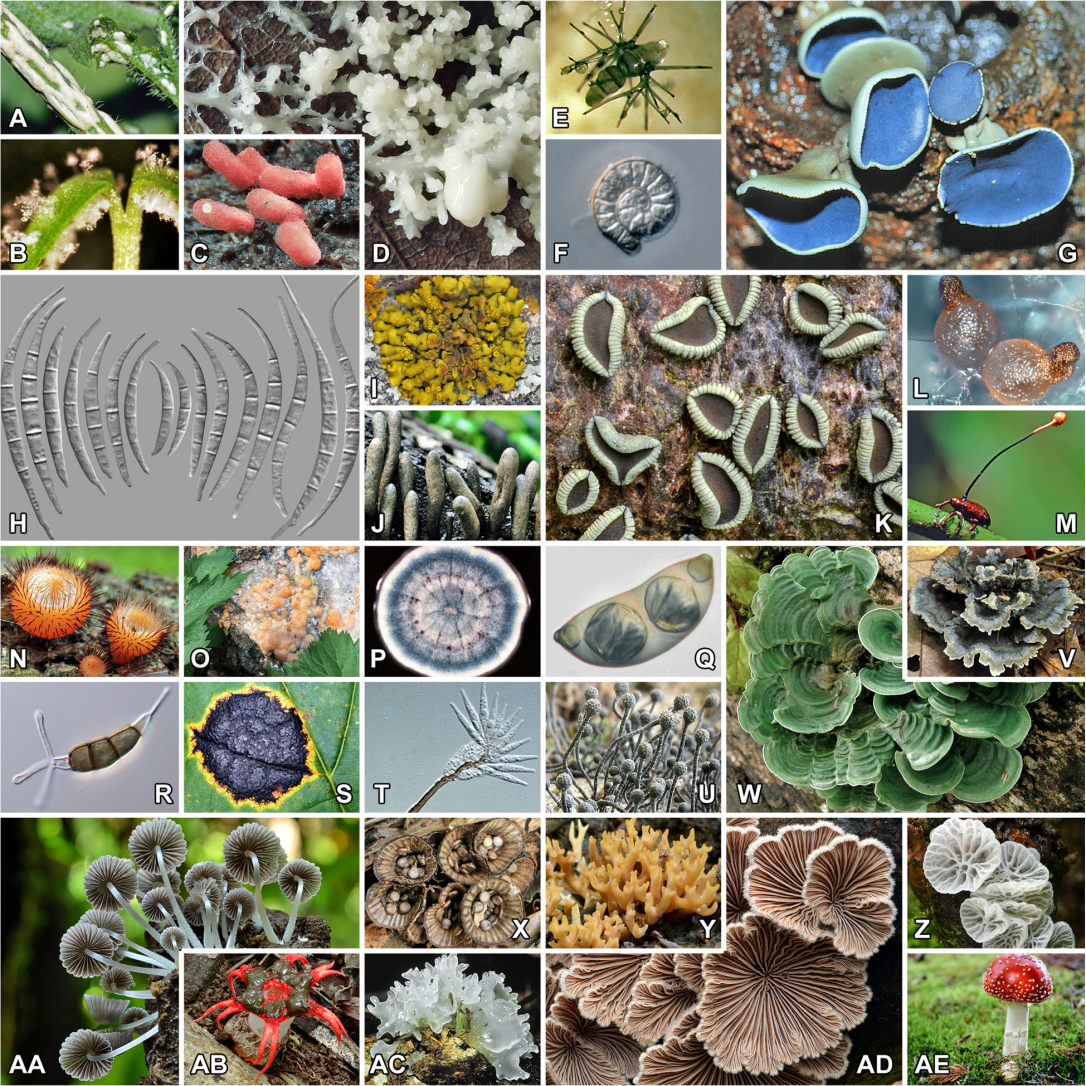
The diversity of *Fungi* and fungal-like organisms is staggering, with between 2.2 to 3.8 million species predicted (Hawksworth and Lücking 2018). Identification tools specifically tailored to each group are indispensable to deal with such richness. **A**–**B**, *Oomycota*; **C**–**D**, *Mycetozoa*; **E**, *Mucoromycota*; **F**–**U**, *Ascomycota*; **V**–**AE**, *Basidiomycota*. **A**, *Albugo candida* (on *Capsella bursa-pastoris*). **B**, *Hyaloperonospora thlaspeos-perfoliati* (on *Microthlaspi erraticum*); for *Oomycota*, *COX1* and *COX2* have been proposed as alternative DNA barcodes (Choi et al. 2015). **C**, *Arcyria denudata*. **D**, unidentified slime mold plasmodium; a portion of the nuSSU, in combination with *COX1* and *TEF1*, has been shown to provide good resolution to delimit species (Schnittler et al. 2017). **E**, *Phycomyces blakesleeanus* (mating). **F**, *Helicoma taenia* (conidium). **G**, *Sorokina caeruleogrisea* (ascomata). **H**, *Fusarium duofalcatisporum* (conidia); secondary DNA barcodes, such as *TEF1*, have been proposed to delimit species in this plant-pathogenic genus (O'Donnell et al. 2015; Al-Hatmi et al. 2016; Xia et al. 2019). **I**, *Placomaronea candelarioides* (thallus). **J**, *Xylaria polymorpha* (stromata bearing ascomata). **K**, *Rhytidhysteron columbiense* (ascomata); this conspicuous saprotrophic genus contains numerous unrecognized species based on ITS (Soto-Medina and Lücking 2017). **L**, *Neocosmospora vasinfecta* (perithecia); this genus is one example of competing solutions to ranking clades in *Fusarium* s.lat. at genus level (Summerell 2019; Sandoval-Denis et al. 2019), a problem that is not resolvable by phylogeny alone (Lücking 2019), but which affects nomenclature of economically important fungi. **M**, *Ophiocordyceps curculionum* (stroma growing out of a weevil). **N**, *Cookeina tricholoma* (ascomata). **O**, basidiomycetous yeast (various members of *Cystofilobasidiales*) efflux on tree stump (Yurkov et al. 2020). **P**, *Aspergillus sydowii* (culture); fungi of this genus can cause aspergillosis in humans and are identified through a combination of DNA barcoding (*TUB2*) and high-resolution melting (HRM) assay (Fidler et al. 2017). **Q**, *Pyrenula subpraelucida* (ascospore). **R**, *Pseudopestalotiopsis ixorae* (conidium); this is another genus for which secondary DNA barcodes (*TEF1*, *TUB2*) have been proposed (Maharachchikumbura et al. 2012, 2014). **S**, *Rhytisma acerinum* (tar spot on *Acer*); recently, a separate, near-cryptic North American species was discovered integrating ITS and biological data (Hudler et al. 1998). **T**, *Macgarvieomyces juncicola* (conidiophore with conidia). **U**, *Batistia annulipes* (stromata). **V**, *Thelephora terrestris* (basidioma). **W**, *Cora imi* (thallus); until recently, this genus was believed to include a single species, but integrative taxonomy combining the ITS barcoding marker and morpho-anatomical and ecological characters recognizes nearly 200 (Lücking et al. 2014, 2017). **X**, *Cyathus striatus* (basidiomata). **Y**, *Ramaria formosa* (basidiomata). **Z**, *Campanella caesia* (basidiomata); based on ITS barcoding data, this presumably European taxon is subcosmopolitan, being also found in North America including Mexico, South America (Colombia; photograph), and Africa (Kenya). **AA**, *Coprinellus disseminatus* (basidiomata). **AB**, *Aseroe rubra* (basidioma). **AC**, *Tremella mesenterica* (basidioma). **AD**, *Schizophyllum commune* (basidiomata); this industrially important taxon includes geographically separated clades based on the IGS (James et al. 2001). **AE**. *Amanita muscaria* (basidioma); according to a three-marker study (ITS, nuLSU, *TUB2*; Geml et al. 2006), this well-known mushroom comprises several cryptic species

Fungi are eukaryotic heterotrophic organisms that mostly grow with elongated, polarized cells (hyphae) or in the form of budding cells (yeast-like), reproducing via meiotic and/or mitotic spores. The fungal lifestyle evolved several times independently in the *Tree of Life* (Fig. 1). The majority of the known species (close to 99%) are true fungi (*Fungi*), whereas about 0.7% represent *Eumycetozoa* and other groups of slime molds in the *Amoebozoa* (supergroup *Amorphea*), and another 0.7% the *Oomycota* in the *Straminipila* (Stephenson et al. 2008; Beakes and Thines 2017; Hawksworth and Lücking 2018; Lado and Eliasson 2017; Willis 2018; Burki et al. 2019; Wijayawardene et al. 2020). *Fungi* rank third among eukaryotic kingdoms in terms of known species richness, with approximately 140,000 species, but the total number has been predicted as between 2.2 and 3.8 million, with a mean of 3 million (Hawksworth and Lücking 2018), with other estimates as low as 700,000 and as high as 12 million (Schmit and Mueller 2007; Blackwell 2011; Vu et al. 2019).

Fungi in the broad sense are ubiquitous in terrestrial, freshwater and marine ecosystems (Dix and Webster 1995; Mueller et al. 2004; Rodriguez et al. 2009; Thines 2014; Asplund and Wardle 2017; Buzzini et al. 2017; Glime 2019; Jones et al. 2019). They carry out important processes as decomposers of organic material contributing to nutrient cycles, parasites controlling host population structure, anaerobic gut mutualists, and mutualists with autotrophic organisms, e.g. the various forms of endophytes, lichens and mycorrhizae. Fungi have economic impact as plant and animal (including human) pathogens, in the biological control of crop pests, in the food and pharmaceutical industry, as edible mushrooms, and are also applied as indicators of environmental health (May and Adams 1997; Nimis et al. 2002; Crawford 2019; Hyde et al. 2019).

Accurate and precise identification of fungi is challenging. Compared to other multicellular eukaryotes, fungi have simple body plans and diagnostic features are generally limited to their sexual and asexual spore-producing bodies, requiring microscopic examination (Beakes and Thines 2017; Nagy et al. 2017; Lücking 2019). Some fungi are only known from vegetative structures, rendering traditional approaches to classification nearly impossible (e.g. Koch et al. 2017, 2018). Precise identification of fungi thus requires removal from their habitat and careful investigation in the laboratory. Exceptions would be well-established taxa which exhibit features discernable in the field, such as the lung lichen, *Lobaria pulmonaria*, the split gill mushroom, *Schizophyllum commune* (Fig. 1ad), or the familiar pathogen causing tar spot on Acer leaves, *Rhytisma acerinum* (Fig. 1s). However, even in such cases, unrecognized cryptic speciation may lead to erroneous phenotype-based identifications, as shown by the recently described *Rhytisma americanum*, which had long been mistaken for *R. acerinum* (Hudler et al. 1998). Even if only a single, morphologically well-defined species is recognized, such as *S. commune*, its genetic structure may be complex (James et al. 2001). This raises questions about species limits and at what level of precision phylogenetic complexity should be recognized taxonomically and, by extension, incorporated in identification tools.

The non-reproductive phase of fungi, typically forming hyphae or budding (yeast-like) cells, or plasmodia in slime molds, is usually cryptic, exhibiting little useful diagnostic information, except for classification attempts based on fungal cultures (Nobles 1965; Stalpers 1978; Pazouki and Panda 2000; Kurtzman et al. 2011). In contrast, many lichen-formers can be identified to species level in the absence of spore-producing structures, due to their persistent thalli (Honegger 2012). Both the higher classification of fungi and the delimitation of species have been notoriously difficult and underwent dramatic changes with the development of molecular approaches (Taylor et al. 2000; James et al. 2006; Hibbett et al. 2007, 2016; Schoch et al. 2009; Crous et al. 2015; Spatafora et al. 2016; Beakes and Thines 2017; Hawksworth and Lücking 2018; Tedersoo et al. 2018a). A further dimension has been added through environmental sequencing, in which the phenotype of detected lineages is unknown except for ecological preferences inferred from metadata (O'Brien et al. 2005; Bellemain et al. 2013; Sirohi et al. 2013; Menkis et al. 2014; Ohsowski et al. 2014; Grube et al. 2017; Lücking and Hawksworth 2018; Thines et al. 2018; Nilsson et al. 2019; Vu et al. 2019; Davison et al. 2020).

Due to the heterogeneity of approaches to fungal taxonomy and the complexity of lineage-dependent evolutionary processes, there are no simple strategies to unambiguously identify fungi (Grube et al. 2017; Steencamp et al. 2018; Inderbitzin et al. 2020). Best practice depends on the group in question and the required level of precision (Raja et al. 2017a). Many macrofungi, some microfungi, and many lichen-formers can be identified using phenotype characters once a reliable taxonomic framework has been established. However, the majority of fungi, especially asexual forms, yeasts and other basal lineages, and those important in fields such as plant pathology and medical mycology, require time-consuming and labour-intensive methods that may include culturing, DNA barcoding and phylogenetic analysis, as well discipline- or taxon-specific approaches, such as physiological profiling (see below).

Two fundamental aspects of identification are accuracy and precision (Vu et al. 2019). To illustrate this concept: accuracy would identify a mushroom as either a true (*Cantharellus cibarius* s.lat.) or a false chanterelle (*Hygrophoropsis aurantiaca*), two unrelated species in different fungal orders. Once verified that the query taxon is a true chanterelle, precision would determine the exact species, as *Cantharellus cibarius* s.lat. Represents several more narrowly defined taxa (Buyck and Hofstetter 2011; Foltz et al. 2013; Leacock et al. 2016). While accuracy is indispensable for identifications, precision depends on the purpose. The latter is particularly critical for legal compliance and regulatory controls, in biosafety regarding clinical diagnosis and subsequent recommendations for disease management of plant and human/animal pathogens, in food security (edible mushrooms, FDA approved species), for quarantine regulations (plant pests), industrial usage, the distribution of dual-use organisms (toxic fungi), or where conservation measures are being administered (Druzhinina et al. 2010; Dahlberg and Mueller 2011; Criseo et al. 2015; Crous et al. 2015; Raja et al. 2017b; Blackwell and Vega 2018; Heim et al. 2018; Frøslev et al. 2019).

## SPECIES: FROM CONCEPTS TO IDENTIFICATION

**Fig. 2:**
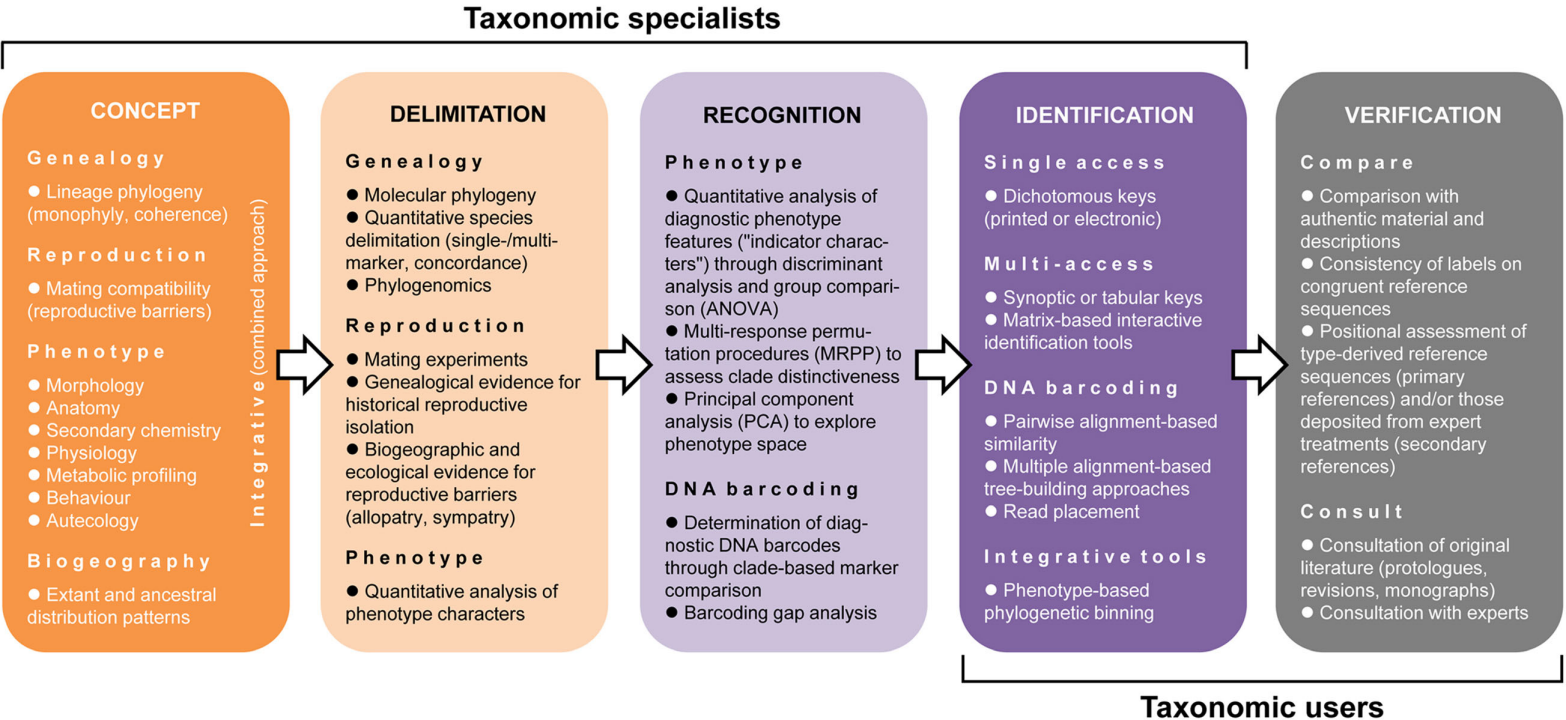
The dependence of fungal identification on species concepts, delimitation and recognition approaches, and the importance of the verification process. Taxonomic specialists typically elaborate the first four steps up to the production of identification tools, whereas taxonomic users apply identification tools and perform verification. The verification process is generally neglected but is of crucial importance for accurate identifications

Often conflated, species conceptualization, delimitation, recognition, identification, and verification involve largely separate approaches, although they logically depend on each other (Fig. 2). Ultimately, for accurate and precise identification in any given fungal group, an underlying concept to delimit species and evaluate their diagnostic characters for recognition needs to be agreed upon before tools for identification and verification can be employed (Harrington and Rizzo 1999; Steenkamp et al. 2018; Inderbitzin et al. 2020).

### Concepts

Across the *Tree of Life*, species concepts are the theoretical basis upon which we recognize and name species; they play, therefore, a crucial role in the development of identification tools. For instance, sexual and asexual morphs in fungi were traditionally named and identified separately under the concept of dual nomenclature. With the advent of DNA sequencing and the ability to match sexual and asexual morphs through sequence data, this approach was no longer necessary, and dual nomenclature was replaced by the concept of “one fungus, one name” (Hawksworth 2011; Taylor 2011; Wingfield et al. 2012; Geiser et al. 2013).

Over 30 concepts have been proposed to delimit species across the *Tree of Life* (Mayden 1997; Zachos 2016; Wilkins 2018). All consider one or several of three fundamental criteria (Fig. 2): genealogical coherence (in particular monophyly), reproductive isolation, and phenotypic distinctiveness (including autecology; e.g. Eyualem and Blaxter 2003). Thus, ‘genealogical concordance species’ and ‘phylospecies’ refer to aspects of genealogy. ‘Morphospecies’ (‘phenospecies’) relate to morphological, anatomical, biochemical or behavioral features, which by extension also include autecology (environmental niche space). ‘Biospecies’ and ‘recognition species’ take into account mating compatibility and reproductive barriers. Special cases include ‘agamospecies’ (asexual lineages not known to reproduce sexually) and ‘nothospecies’ (of hybrid origin). Some concepts integrate criteria of genealogy, phenotype and/or reproduction, such as ‘cohesion species’ and ‘evolutionary species’, whereas others aim at the highest possible resolution, e.g. ‘evolutionary significant unit’ and ‘least inclusive taxonomic unit’ (Moritz 1994; Wilkins 2018). As a result, different concepts may result in delimiting species of different size and complexity (Agapow et al. 2004; Taylor et al. 2006; Yurkov et al. 2015a), which may confound users employing identification tools based on “competing” species concepts.

Hawksworth (1996: 32) pragmatically defined fungal species as “*... groups of individuals separated by inheritable character discontinuities and which it is useful to give a species name to ...*”. Since inheritable character discontinuities can only be assessed by simultaneous analysis of phylogenetic relationships and clade-based phenotype variation, this definition is largely congruent with ‘phylogenetic taxon species’ (Eldredge and Cracraft 1980; Nelson and Platnick 1981; Wilkins 2018). It is also in agreement with the ‘consolidated species concept’ of Quaedvlieg et al. (2014). Other terms that have been coined for this approach are the polyphasic species concept and integrative taxonomy (Vandamme et al. 1996; Yeates et al. 2011; Goulding and Dayrat 2016; Lücking 2019; Vinarski 2019). Fungi are no exception to the notion that species have individual evolutionary histories, and so aspects of their genealogical coherence, reproductive isolation and phenotypic distinctiveness may differ. This implies that there is no single, universal approach to species delimitation and consequently for species identification.

The diversity of trophic and reproductive strategies of fungi and their often complex lifecycles add further complications. What is perceived as phenotypically distinct entities may be manifestations of one and the same fungus, often representing sexual *versus* asexual forms (Kendrick 1979; Aoki and O'Donnell 1999; Covert et al. 2007; Wingfield et al. 2012; Rossman et al. 2016; Tanaka and Honda 2017; Tanney and Miller 2017). Exemplar cases are the rust fungi (Aime et al. 2018; Kolmer et al. 2018), which can produce up to seven morphologically and functionally distinct types of spores (Bruckart et al. 2010). So-called “species pairs” in lichens may belong to a single taxon or exhibit complex phylogenies in which the mode of reproduction is not necessarily diagnostic (Mattsson and Lumbsch 1989; Kroken and Taylor 2001; Crespo and Pérez-Ortega 2009; Crespo and Lumbsch 2010; Messuti et al. 2016). The same lichen fungus can also form different vegetative structures depending on the associated photobiont, resulting in strikingly disparate “photosymbiodemes” (Armaleo and Clerc 1991; Högnabba et al. 2009; Moncada et al. 2013).

### Delimitation

While it is difficult to decide *a priori* which approach to species delimitation best applies to a given fungal group, biological and phenotypic aspects have practical and theoretical limitations. The phenotypic approach is limited due to the simplicity of fungal features, such as spore characters, as homoplasious evolution and a disjunct between the timing of genealogical and phenotypic separation may lead to phenotypically cryptic taxa (Carriconde et al. 2008; Lumbsch and Leavitt 2011; Hyde et al. 2011; Balasundaram et al. 2015; Hawksworth and Lücking 2018). Perceived lack of phenotypical divergence can also stem from failure to properly observe diagnostic characters (Moncada et al. 2014; Lücking et al. 2017; Merényi et al. 2017). This is particularly obvious in microfungi; for instance, Johnston et al. (2017) showed that 23% of historical *Phoma* cultures determined based on phenotype had been misidentified.

Reproductive isolation is emphasized as a key trait in the biological species concept (Mayr 1942). In the original description of *Neurospora,* species were recognized in part based on mating compatibility (Shear and Dodge 1927), long before the term “biological species” was first applied. However, more often than not it is difficult to assess reproductive isolation in fungi, and this approach is largely restricted to select taxa including model organisms (Yarden 2016). Mating is inherently cryptic and often complex, involving the fusion of minute gametangial elements, an event rarely observed in nature or even in the laboratory (Kück and Pöggeler 2009; Ni et al. 2011; Ropars et al. 2016; Bruns et al. 2018; Nagel et al. 2018; Li et al. 2020a). There are challenges in the interpretation of mating experiments, as failed mating does not necessarily prove two lineages to represent different species. Sexual reproduction of biotrophic lineages depends on the availability of a suitable host, the absence of which may result in unsuccessful mating tests (Cai et al. 2011; Yurkov et al. 2015b). Successful mating can also occur through homothallism or through hybridization between phylogenetically and morphologically distinct species (Sun et al. 2014). Additionally, many fungi do not appear to reproduce sexually, having lost this ability during evolution (Seifert and Gams 2001; Shenoy et al. 2007; Hyde et al. 2011), although it can sometimes be induced under laboratory conditions (O'Gorman et al. 2009). Given these shortcomings, historical reproductive isolation can be documented through a genealogical concordance phylogenetic species recognition (GCPSR) approach, which identifies shared genealogical partitions between lineages across multiple loci as evidence of isolation (Taylor et al. 2000). While this approach has been applied in fungi (Koufopanou et al. 1997; Geiser et al. 1998, 2007; O'Donnell et al. 2004; Aoki et al. 2019), it does not necessarily identify intrinsic reproductive barriers as the basis for a lack of genetic exchange, and it may reveal populations rather than species (Sukumaran and Knowles 2017). Another approach is the analysis of mating genes to predict sexual compatibility in fungi (Sun et al. 2014, 2019; Yurkov et al. 2015b; Diaz-Valderrama and Aime 2016). In general, reproductively incompatible groups within phenotypically defined species tend to correlate fairly well with phylogenetically supported lineages, as observed in *Neurospora* (Dettman et al. 2003a, b), *Cryptococcus* (Passer et al. 2019), *Fusarium* (Aoki and O'Donnell 1999; O'Donnell et al. 2000), *Penicillium* (López-Villavicencio et al. 2010), *Lentinellus* (Miller and Methven 2000), and *Pleurotus* (Vilgalys and Sun 1994). However, over-reliance on Mendelian-inherited traits may lead to incongruences between phenotypically and phylogenetically defined species (Aime 2004).

Because of these challenges, modern fungal taxonomy emphasizes a genealogical approach, including single- or concatenated multi-gene phylogenies, genealogical concordance, and phylogenomics. The main advantage of this approach is that it can be explored within an explicit hypothetical framework, and phenotypic characters can be placed a posteriori into an evolutionary context. Another advantage is the large number of characters analyzed: whereas phenotype matrices may at best contain a few hundred characters and often less than one hundred, sequence data range from several hundred (single-marker) to thousands (multi-locus) to hundreds of thousands or more (phylogenomics) of sites. However, even with molecular data, difficulties arise from a lack of understanding of evolutionary processes, which are not always discernible in a phylogeny. For instance, recently emerging species may not resolve through reciprocal monophyly (Cunnington et al. 2005; Goodman et al. 2009; Przyboś et al. 2015; Lachance 2016; Leavitt et al. 2016; Liu et al. 2017). These problems are further compounded by often improper taxon selection for molecular analysis, as the most closely related sequences may not be included in the data set or the closest relatives may not have been sequenced. For instance, Evans et al. (2002) suggested placement of the frosty pod rod, *Moniliophthora roreri*, an important pathogen on cacao, in the genus *Crinipellis*, based on the notion that its ITS sequence blasted most closely to *Crinipellis perniciosa*. Subsequent phylogenetic analysis, however, demonstrated that the latter was not a genuine *Crinipellis* but formed a separate generic lineage together with *Moniliophthora roreri* in *Marasmiaceae* (Aime and Phillips-Mora 2005; Kerekes and Desjardin 2009; Evans 2016; Niveiro et al. 2020).

Whole-genome level approaches are increasingly employed in fungi to surmount issues of resolution and support in single- and multi-marker studies (Gladieux et al. 2015; Magain et al. 2017; Lorch et al. 2018; Kobmoo et al. 2019; Morin et al. 2019; Haridas et al. 2020). For prokaryotes, the computationally inexpensive assessment of average nucleotide identity (ANI) has proven popular, although maximum-likelihood methods are also being applied (Parks et al. 2018). Multiple prokaryotic genomes are readily available including from type material (Konstantinidis and Tiedje 2005; Ciufo et al. 2018). Another genome-based approach to resolve species complexes in prokaryotes is Percentage of Conserved Proteins (POCP) analysis (Qin et al. 2014; Martinez-Romero and Ormeño-Orrillo 2019; Peix et al. 2019; Wittouck et al. 2019; Rensink et al. 2020), a method that has now also been implemented in fungi (Wibberg et al. 2020). These strategies are still impractical for broad exploration of fungal diversity, as the accurate analysis of fungal genomes is a time-consuming process and sampling remains sparse, although high quality genomes requiring fewer analytical resources may soon become available with improved third generation sequencing techniques, such as PacBio Sequel and Oxford Nanopore Technologies (Tedersoo et al. 2018b; Loit et al. 2019; Stadler et al. 2020; Wibberg et al. 2020). For difficult species complexes, sequencing of restriction site-associated DNA markers (RADSeq) is another emerging approach in fungal taxonomy (Grewe et al. 2017, 2018; Salas-Lizana and Oono 2018).

Integrative taxonomy attempts to combine as much evidence as possible from genealogical, biological, phenotypic and other approaches to delimit species (Aime 2004; Will et al. 2005; Yang and Rannala 2010; Padial et al. 2010; Udayanga et al. 2014; Haelewaters et al. 2018; Kruse et al. 2018a). The different approaches are thereby not competitive but components of a holistic strategy. Species hypotheses are normally established using phenotypic characters and, where possible, tested by reconstructing the underlying genealogy through molecular phylogeny. This strategy is now often inverted, by detecting novel lineages through phylogenetic analysis and then evaluating these through correlation with phenotypic characters (Millanes et al. 2011; Liu et al. 2015; Lücking et al. 2017; Kruse et al. 2018b). The phenotype has not become obsolete, but forms an important component of integrative taxonomy, including by extension aspects of autecology, physiology, and biochemistry. The phenotype also remains important when evaluating diagnostic characters for identification tools and in cases where it has not been possible to obtain sequence data. Biogeography represents an additional dimension assessed independently of phenotype and ecology and is often used to recognize phenotypically cryptic, allopatric lineages (James et al. 2001; Yurkov et al. 2015a; Sánchez-Ramírez et al. 2015; Lücking et al. 2017).

### Recognition

Quantitative species delimitation analyzes topological aspects of one or several phylogenetic trees, such as genetic distance (branch length patterns), support and concordance (Ence and Carstens 2011; Lim et al. 2011; Fujita et al. 2012; Puillandre et al. 2012; Zhang et al. 2013; Fujisawa et al. 2016). In contrast, recognition subsequently detects diagnostic features that allow lineages delimited through phylogeny to be recognized (Somervuo et al. 2006; Trifa et al. 2008; Kruse et al. 2018a, b). Delimitation may be based on a broad set of data, including whole-genome data, whereas lineages thus delimited may be recognized by few diagnostic features, either phenotypic or through DNA barcodes. For certain fungi, including molds and yeasts, diagnostics may be derived from physiological profiles as determined by VITEK or API systems, high-resolution melting (HRM) assays, and proteomics via MALDI-TOF (Buesching et al. 1979; Fenn et al. 1994; Kurtzman 2006; Gazis et al. 2011; Nenoff et al. 2013; Yurkov et al. 2015a, b; Fidler et al. 2017; Patel 2019; Passer et al. 2019). Species delimitation and recognition are often confounded, and “species recognition approaches” often refer to species delimitation (e.g. Dettman et al. 2003a, b; Geiser et al. 2007; Grünig et al. 2007).

Single phenotype characters or DNA barcoding markers may provide reliable discrimination in many fungi. However, often a combination of characters or markers is needed to achieve the desired accuracy and precision, sometimes incorporating character weighting (Berger et al. 2011; Dupuis et al. 2012; Krüger et al. 2012; Kruse et al. 2018b; Liu et al. 2015; Yurkov et al. 2015b). Another conceptual difference between species delimitation and recognition is that diagnostic characters are not necessarily used for delimitation; typically, delimitation is based on molecular phylogeny, whereas recognition relies on quantitative (statistically tested) analysis of phenotypic characters mapped *a posteriori* onto phylogenetic trees, the desirable standard approach not only in fungal taxonomy.

### Identification

Following species delimitation and recognition, a critical step is needed to enable identification: the generation of effective identification tools that synthesize the available information (Fig. 2). These may range from traditional dichotomous to computerized interactive keys based on the phenotype, to molecular identification, such as DNA barcoding, or a combination of various methods (Druzhinina et al. 2005; Coleman et al. 2010; Reginato 2016; Attigala et al. 2016; Smith Jr 2017; Nguyen et al. 2017; Van Sinh et al. 2017; Tofilski 2018). Recent developments in plant taxonomy include machine-learning tools to evaluate phenotype features (Hernández-Serna and Jiménez-Segura 2014). This approach works rather well in features with a particular architecture, such as leaves, enabling powerful applications, such as *Leafsnap* and *Leafnet* (Kumar et al. 2012; Barré et al. 2017; Kress et al. 2018). For fungi, image-based identification is challenging, since quantitative morphometry cannot usually be applied, although there might be some use in the detection of plant diseases (Pujari et al. 2015; Heim et al. 2018).

Providing effective identification tools is one of the fundamental tasks of taxonomists, not only in mycology. Based on available phylogenetic treatments, taxonomic experts are encouraged to employ state-of-the-art methods to assemble comprehensive data sets for diagnostic characters, which allow the creation of interactive and/or automatically derived dichotomous or synoptic keys for a given group (e.g. Rambold 1997; Zambonelli et al. 2000; Druzhinina et al. 2005; Triebel et al. 2016; Nguyen et al. 2017). *MycoBank Polyphasic Identifications Databases* provides links to identification tools for various groups of fungi [http://www.mycobank.org/DefaultInfo.aspx? Page = polyphasicID]. For plant pathogens, the USDA *Fungal Databases* website [https://nt.ars-grin.gov/fungaldatabases] is also helpful (Farr and Rossman 2020).

Identification tools and descriptions of new taxa should be freely accessible. The latter is possible through registration of fungal names in *MycoBank*, *Index Fungorum* or *Fungal Names*; the deposition of images is not obligatory but strongly recommended. Open access options for identification tools often conflict with the needs for publication impact and the inflated costs for open access models. In such cases, a practical remedy is to post pre-publication manuscripts in a free repository, such as bioRxiv (Sever et al. 2019), so that users can freely access the information while citing the original paper. Unified digital protologues with semantic standardization can be a further step towards automated collection, structuring and analysis of taxonomic data, based on both specimens and species (Kilian et al. 2015; Triebel et al. 2016; Plitzner et al. 2019; Dallwitz et al. 2020). However, this approach is challenging due to terminological ambiguity and the large set of characters required to cover all fungi, only a fraction of which is typically used in a particular lineage.

### Verification

**Fig. 3:**
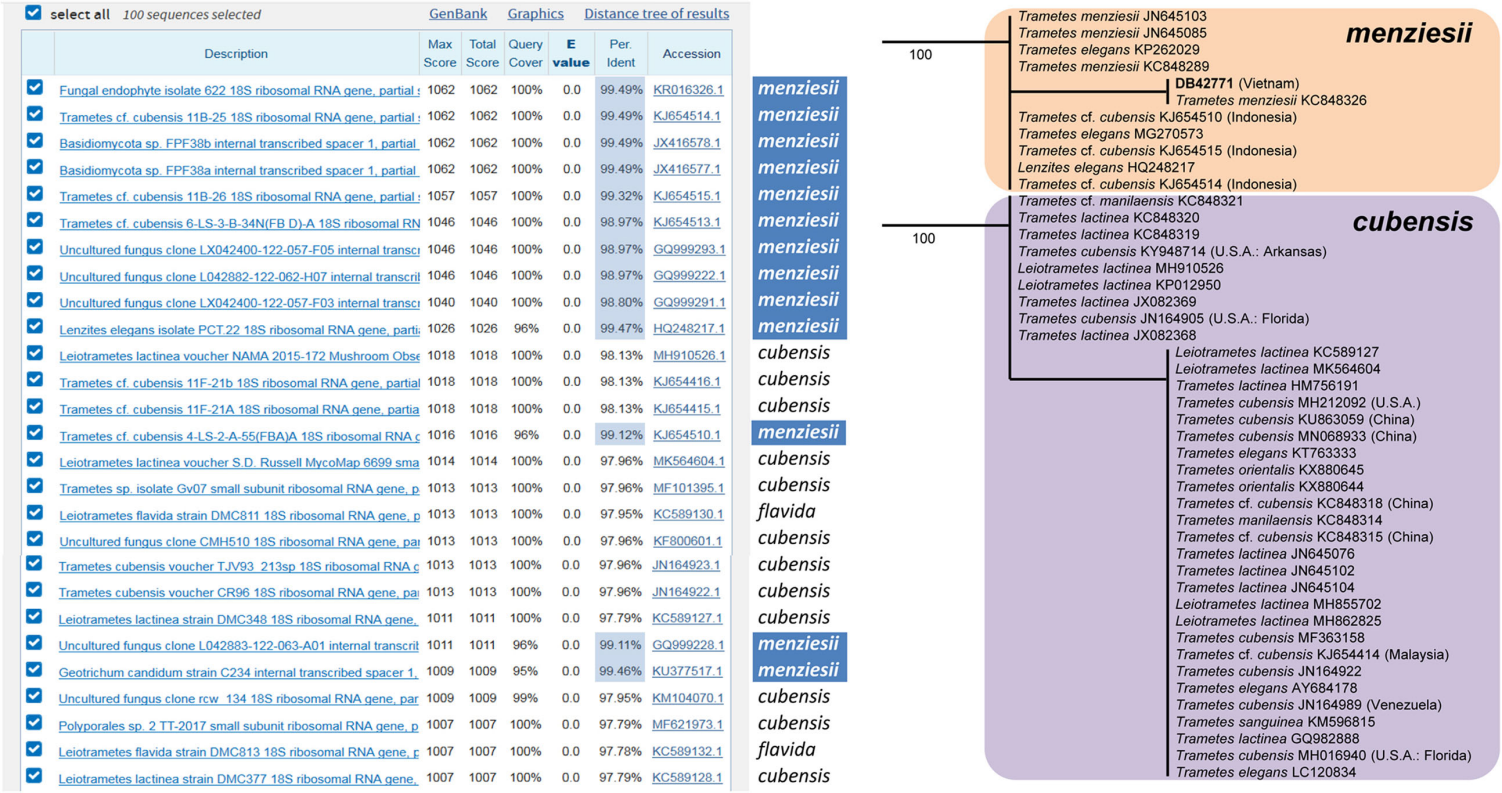
Comparison of BLAST-based (pairwise alignment) vs. tree-based (multiple alignment) identification of a target fungal ITS sequence (DB42771, Vietnam; see Lücking et al. 2020)**.** BLAST (both blastn and megablast) initially suggested *Trametes cubensis* or *Leiotrametes lactinea* to be the most likely identification: the label ‘cf. *cubensis*’ had the three highest named BLAST hits and appeared six times among the top ten named hits. Yet, multiple alignment-based phylogenetic analysis placed the target sequence in a clade corresponding to *T. menziesii*, described from Indonesia. Apart from demonstrating the shortcomings of BLAST identifications, this example illustrates numerous problems with reference sequence labeling, including wrongly identified sequences and confusion about species and even genus concepts and nomenclature (Lücking et al. 2020). A user not aware of such issues would not be able to obtain a reliable identification using BLAST only, whereas the alignment-based phylogenetic approach followed by a verification process provided an accurate result in this case. Notably, two remedies would substantially improve BLAST identification results: (1) correct labeling of the reference sequences through third-party annotations (middle column), plus (2) sorting BLAST results by percentage identity (highlighted values)

Users often uncritically accept identifications achieved with a given tool, although the identification process may lead to a wrong name. This happens not only in phenotype-based approaches but also with molecular identifications, when reference sequences are incorrectly labeled or follow an inappropriate taxonomic concept, or through uncritical use of pairwise similarity-based approaches such as BLAST (see below and Fig. 3). Different BLAST algorithms (megablast, discontinuous megablast and blastn) can yield different matches, depending on the length of the query and/or reference sequences, what score is observed, and whether sequences of the underlying marker, such as the ITS, were deposited in their entirety or separately, e.g. ITS1 versus ITS2 (Altschul et al. 1990; Camacho et al. 2009; Nilsson et al. 2008; Blaalid et al. 2013; Tedersoo et al. 2015; Madden et al. 2019; Větrovský et al. 2020). This underlines the importance of the verification process. Verification must thereby go beyond the data used for identification, to avoid circular conclusions (Lindahl et al. 2013; Hart et al. 2015; Vu et al. 2019). Unfortunately, verification is impractical or next to impossible for massive amounts of data, such as in environmental metabarcoding approaches, which consequently require trade-off between speed and accuracy (see below).

Verification steps are manifold but largely depend on the nature of diagnostic characters and whether phenotypic or molecular annotations are being used. For phenotype-based identifications, verification relies on consultation of original descriptions and examination of authentic specimens (including cultures) and/or imagery, including digitized type material in repositories, such as *JSTOR Global Plants* (Ryan 2013, 2018) or the *Mycology Collections Portal* (Miller and Bates 2017). *Species Fungorum* [http://www.speciesfungorum.org], *MycoBank* [http://www.mycobank.org], *The Faces of Fungi* [http://www.facesoffungi.org], *The Yeasts Trust Database* [http://www.theyeasts.org], USDA *Fungal Databases* [https://nt.ars-grin.gov/fungaldatabases], the *Biodiversity Heritage Library* [https://www.biodiversitylibrary.org], *Cyberliber* [http://www.cybertruffle.org.uk/cyberliber], and *Google Scholar* [https://scholar.google.com], are excellent tools to obtain information about original and other taxonomic literature, often with direct links to available sources (Crous et al. 2004; Robert et al. 2013; Jayasiri et al. 2015; Farr and Rossman 2020; Boekhout et al. 2020). Confirmation by specialists is another option, which of course requires the continued existence of a sufficient number of taxonomic experts (Lücking 2020).

Although often neglected, phenotype-based verification is also indispensable for sequence-based identifications. To facilitate this process, it is recommended to generate digitally accessible images of sequenced voucher material and deposit the material in registered fungaria (Thiers 2018), with links between sequence data, voucher information, and digital imagery (Krah et al. 2019). Other possibilities include improving the accurate annotation of vouchers enforcing structured information for biorepositories (Güntsch et al. 2017; Sharma et al. 2018), especially during name registration, publication and sequence submission to GenBank and its partners in the *International Sequence Database Collaboration* (INSDC). The AJOM fungal notes series publishes new collections of known species with sequence data (Hyde et al. 2020) in a novel format to emphasize the importance of such contributions. The data with imagery is also placed online in websites developed for specific groups (Jayawardena et al. 2019; Pem et al. 2019; Li et al. 2020b).

Entirely sequence-based verification can be achieved through multiple alignment-based phylogenetic analysis and checking the placement of authentic reference sequences, in particular those based on type specimens. BLAST offers the option to limit hits to “Sequences from type material” (Federhen 2015), but since their number is still low and biased towards particular lineages, this option is currently only of theoretical use for broad fungal surveys. If type-derived sequences are not available, curated sequence databases can be consulted for vetted non-type reference sequences, such as UNITE (Abarenkov et al. 2010; Kõljalg et al. 2013, 2019; Nilsson et al. 2019), NCBI RefSeq (Targeted Loci) (Schoch et al. 2014), the various group-specific sources linked through *MycoBank* BioloMICS Sequences (Robert et al. 2013), or specialized databases for plant and animal/human pathogens, such as Q-Bank and the International Society of Human and Animal Mycology (ISHAM) ITS reference DNA barcoding database (Bonants et al. 2013; Irinyi et al. 2015). Third-party annotations in primary repositories, such as GenBank, both directly and as push-back mechanism from curated databases (Fig. 3), would also be valuable. Alternatively, NCBI RefSeq (Targeted Loci) could be extended to include additional sequences from reference material in public collections, e.g. non-type sequences vetted through multi-locus phylogenetic analysis by third parties in a publication. Another option would be to implement a simple, third-party annotation system that links three unique identifiers: (a) GenBank accession of sequence to be annotated; (b) MycoBank/Index Fungorum/FungalNames registration number of the name representing the correct identification; (c) DOI of the publication that documents the correct identification. Such a flat table could be centrally curated and incorporated in automated identification pipelines.

Interactive polyphasic identification tools such those based on DELTA IntKey, *MycoKeys*, *DiscoverLife* IDnature guides, *Dryades* KeyToNature or *MyCoPortal* keys offer the possibility to obtain verification feedback through the identification process about the taxa remaining in a pool, after selecting a set of characters and states (Dallwitz 1993; Han et al. 2010; Nimis et al. 2012; Lücking and Pickering 2020; Miller and Promputtha 2020a, b; Miller et al. 2020a, b). Phenotype-based phylogenetic binning (Berger et al. 2011) not only integrates molecular and phenotype data but also allows the establishment of automated identification tools, such as *PhyloKey*, which compute bootstrap support values as reliability measures for phenotype-based identifications on a molecular phylogenetic backbone, thus incorporating an automated verification step (Lücking et al. 2016). Assembling the underlying data matrices for such approaches is time-consuming, but it results in directly verifiable identifications and a structured, more objective, reproducible identification process.

## CHALLENGES WITH REGARD TO UNAMBIGUOUS IDENTIFICATION OF FUNGI

### Universal, unambiguous identification of fungi: does one size fit all?

Phenotypically cryptic speciation and convergent evolution are frequent in fungi (Crespo and Pérez-Ortega 2009; Cai et al. 2011; Moncada et al. 2014; Balasundaram et al. 2015; Jayawardena et al. 2016; Liu et al. 2017; Kruse et al. 2018b). Formal taxonomy that recognizes cryptic species may appear impractical because the molecular tools necessary for precise identification are out of reach for many users. However, phylogenetic distinctiveness of lineages should not be dismissed because methods for their detection are not readily available (Hawksworth 2016). For each group of fungi, approaches to identification have to be cognizant of the current species concept established for that group, the methods to evaluate that concept, and the required level of precision. Lack of accuracy of fungal identifications cannot be excused by the lack of adequate tools, and so the availability of tools determines which fungi can be studied. However, lack of molecular tools can be partially balanced by expertise: talented and knowledgeable mycologists may provide more accurate species identifications through non-molecular approaches than unexperienced users do through DNA-based identifications.

Ecological studies in fungi often emphasize statistical data analysis over accuracy and precision of taxon identifications. The common practice of identifying operational taxonomic units (OTUs) to only higher taxa (genus, family, order) should be avoided, unless this is the desired level of precision, justified by the objectives and underlying assumptions, or in environmental metabarcoding when no close relatives have been sequenced (Caporaso et al. 2010; Huson et al. 2011; Veresoglou et al. 2013; Kemler et al. 2017; Kahlke and Ralph 2019). This also includes the use of uncritically adopted generic names in polyphyletic circumscriptions and listing informally named morphospecies without proper reference allowing their recognition in another context. The obvious solution lies in interdisciplinary collaboration (Öpik and Davison 2016; Grube et al. 2017). However, this is rarely realized, one of the reasons why the importance of taxonomy is not broadly acknowledged (Seifert et al. 2008; Lücking 2020). We recommend ecologists, plant pathologists and researchers in other fields of study that rely on fungal taxonomy and associated data (e.g. species traits such as functional spore morphology; e.g. Aguilar-Trigueros et al. 2019) to collaborate with taxonomists, and we encourage taxonomists to make themselves available for such collaborations. After all, this is one of the core duties of taxonomic experts, but it also requires continuous support for this field of study (Lücking 2020).

In cases of DNA-based identifications, users often blindly rely on the presumed accuracy of reference data (see below), and there is usually no consultation with taxonomic expertise. Another issue is the habit of citing sequence accession numbers as “sources” of identifications, while ignoring the underlying taxonomic work that let to the deposition of these valuable reference sequences in the first place. Looking up and citing these works is an important step in quality filtering of reference sequences and to some extent can replace taxonomic expertise when assessing results of DNA-based identifications. In environmental metabarcoding approaches, taxonomic expertise is unfortunately largely fruitless due to the absence of physical voucher specimens. Also, since metabarcoding typically encompasses a broad diversity of higher taxa (Tedersoo et al. 2014; Davison et al. 2018; Ruppert et al. 2019), it is impossible to achieve high levels of accuracy and precision for species identifications across all lineages, but there are alternative strategies to obtain reliable results in such studies (see below).

For plant- and animal/human-pathogenic or industrial fungi, a high level of taxonomic precision is required that cannot usually be achieved by phenotypic identifications. Instead, DNA barcoding or specific diagnostic testing and profiling have become indispensable (Criseo et al. 2015; Crous et al. 2015, 2016; Irinyi et al. 2015; Heim et al. 2018; Hoang et al. 2019). The emerging multi-drug resistant yeast *Candida auris* is one example of a fungus misidentified by phenotypic tools (Chatterjee et al. 2015; Lockhart et al. 2017). Identification of quarantine pests, such as *Phyllosticta citricarpa*, the causal agent of Citrus Black Spot disease (Guarnaccia et al. 2017), is another example where a particular molecular marker should be employed, as recommended by the Q-Bank of the *European and Mediterranean Plant Protection Organization* (EPPO; Bonants et al. 2013). Manuals help to select proper genetic markers for identification of plant pathogenic, clinical and food-borne fungi (Marin-Felix et al. 2019; Samson et al. 2019; de Hoog et al. 2020). In certain cases, the species level may not be sufficiently precise, and identification of particular lineages or strains may be required (Pegg et al. 2019).

Because of these issues, presently there is no single identification method that would universally apply to all fungi and be broadly available to users.

### Reference data: the bread and butter of identification tools

Identification tools are only as good as the reference data behind them. For phenotype-based keys, taxa under all published names in a group need to have been studied, usually as the result of monographic treatments or revisions. Where no keys are available, it is necessary to consult published descriptions and reference specimens, an often painstaking, yet indispensable, approach that is nowadays facilitated by digital repositories (see above). The accessibility of reference material, both physically and virtually, is crucial in this process. Ideally, a broad array of characters needs to be quantitatively analyzed to determine those most effective for identification (e.g., Sieber et al. 1998).

**Fig. 4:**
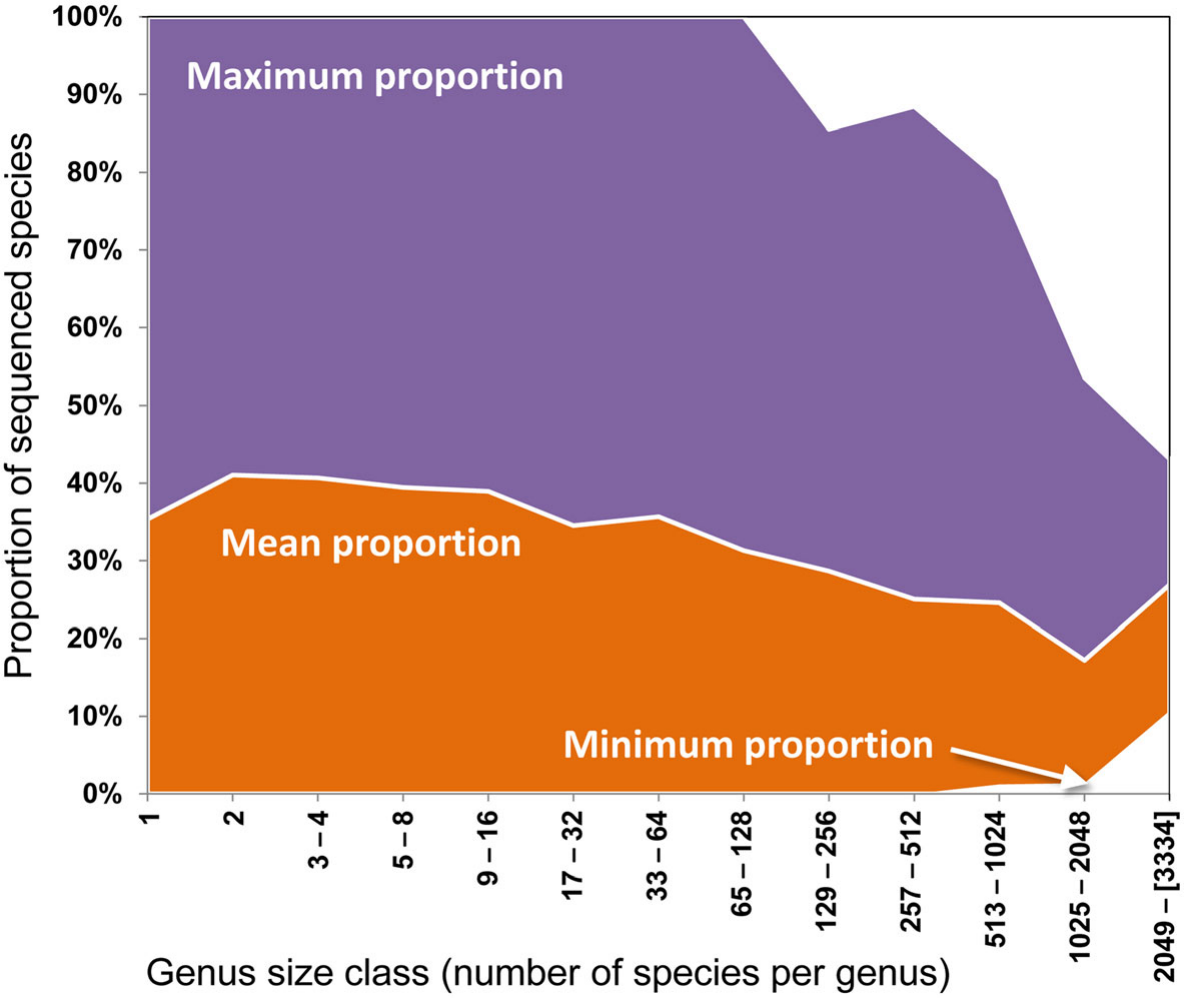
Proportion of species with sequence data compared to total number of species per genus known in fungal genera, based on integration of the NBCI taxonomy and *Species Fungorum*. The mean proportion varies between 40% in species-poor genera and as little as 20% in species-rich genera. At least some species-poor to moderately diverse genera have all species sequenced, whereas many others are devoid of sequenced species. In more diverse genera, the maximum proportion of sequenced species sharply drops as a function of species richness, but also the minimum proportion increases, meaning that all large genera have at least some species sequenced but are consistently incomplete

For DNA barcoding, completeness of reference sequences is critical, but unfortunately still rudimentary for many fungi, especially for species-rich genera (Fig. 4). Currently, sequence data exist for ca. 45,000 named fungal species, most of these including ITS. This corresponds to about 30% of known species, but only 6% when assuming a minimum of 700,000 species (Schmit and Mueller 2007) and 1–2% when considering 2.2–3.8 million (Hawksworth and Lücking 2018). Closing this substantial gap must be a priority of the mycological community (Osmundson et al. 2013). Curated databases, such as UNITE, MaarjAM, ISHAM DNA barcoding, NCBI RefSeq (Targeted Loci) and CBS/WI (Öpik et al. 2010, 2014; Kõljalg et al. 2013, 2019; Schoch et al. 2014; Irinyi et al. 2015; Vu et al. 2016, 2019) play an important role in this endeavor. UNITE features close to 2.5 million curated fungal ITS sequences, corresponding to over 100,000 species hypotheses at a default threshold of 98.5% identity. However, most of these species hypotheses remain unnamed. Many newly published species names remain unrecorded in public sequence databases by failure of submitters to update their records, a problem that can be remedied by standardized keywords and/or listing of type-based DNA barcode accessions in taxonomic treatments (Lücking et al. 2017; Schoch et al. 2017).

A common misconception in DNA barcoding is the assumption that existing reference data provide a definitive answer, either in species identification or to establish whether a taxon is new. Such an approach will fail when reference data are incomplete or sequences are improperly labeled (Nilsson et al. 2006). Methods such as reference OTU picking, implemented in QIIME and other pipelines (Caporaso et al. 2010; Bik et al. 2012; Rideout et al. 2014; Cline et al. 2017), are highly sensitive to the quality and scope of reference databases, although open reference OTU picking allows recognition of query sequences that do not have close reference matches. Potential error is also hidden in what has been called *last* (*lowest) common ancestor* (LCA) analysis in analytical packages, such as MEGAN, QIIME and BASTA (Caporaso et al. 2010; Huson et al. 2011; Kahlke and Ralph 2019), an approach commonly used in environmental metabarcoding of fungi (Majaneva et al. 2015; Miller et al. 2016; Sinha et al. 2017; Anslan et al. 2018). This algorithm identifies the most similar sequences in a reference database and returns the highest shared taxonomy level obtained from the corresponding NCBI taxonomy. For instance, if the five best hits all represent (a) the same species, (b) the same genus but different species, or (c) the same family but different genera, the query sequence is identified either to the level of (a) species, (b) genus, or (c) family. The accuracy and precision of this approach is determined by the sequence labels, as well as how similar the closest hits are to the query sequence. In the case of the above barcoding example of *Trametes menziesii* from Vietnam (Fig. 3), LCA would return *Basidiomycota* (phylum) as the highest level of precision, even if the underlying data would allow an identification to species. Excluding all undetermined sequences, the best hits would include the genus names *Trametes*, *Lenzites* and *Leiotrametes* and hence return the family *Polyporaceae* as highest level of precision. Curated databases, such as UNITE permit the use of the species hypothesis identifier as highest level of precision, but this is cumbersome in the interpretation of massive amounts of data.

For phenotype-based identifications, a frequent error is the use of improper identification tools which may either be outdated, incomplete, or geographically inappropriate. For a given group in a geographic region, proper identifications tools are often not available and one has to rely on “alien” sources. In such cases, identifications should at best be considered initial approximations. Unfortunately, checklists and digital specimen repositories contain numerous presumably widespread fungal species because a tool established for a particular region has been used to identify taxa elsewhere. High quality treatments, such as *Mushrooms of North America* (Phillips 1991) and *Lichens of North America* (Brodo et al. 2001) have become popular identification tools for users across the world (e.g. Ecuador, macrolichens: González et al. 2017; Brazil, ectomycorrhizal fungi: Giachini et al. 2000; Israel, *Acarospora* lichens: Temina et al. 2005; India, edible mushrooms: Singh et al. 2017). However, identifications based on such “alien” sources have to be treated with caution.

## CAVEATS OF THE ITS AS UNIVERSAL DNA BARCODING MARKER IN FUNGI

Molecular identification is rapidly becoming a major tool in fungal taxonomy, due to its universal applicability, speed, and the presumption that it replaces taxonomic expertise, making this approach broadly applicable in many fields of mycology (Yahr et al. 2016). In environmental metabarcoding, it is in fact the only tool available (Epp et al. 2012; Toju et al. 2012; Hibbett et al. 2016; Miller et al. 2016; Lücking and Hawksworth 2018; Tedersoo et al. 2018b; Ruppert et al. 2019). The latter issue is of particular importance, as data from environmental studies grow exponentially. The already outdated number of fungal ITS reads in the SRA (9,762,039,423 as of January 2019) surpasses the number of fungal ITS sequences accessioned in GenBank (1,367,715 as of March 2020) by a factor of more than 7000 (currently likely over 10,000). Six years ago, this ratio was 20:1 and just two years ago, it had increased to 1000:1 (Lücking and Hawksworth 2018). Many developments in this context work towards automated pipelines which rely principally on sequence similarity assessment based on the idea of a universal fungal barcoding marker, such as the ITS (Majaneva et al. 2015; Sinha et al. 2017; Anslan et al. 2018).

Following the initial idea of universal DNA barcoding (Gressel and Ehrlich 2002; Hebert et al. 2003; Seifert et al. 2007; Meusnier et al. 2008; Begerow et al. 2010), the fungal ITS was proposed as the first universal fungal barcoding marker, being mostly easily amplified and sequenced and providing acceptable resolution in a wide range of taxa (Nagy et al. 2012; Schoch et al. 2012; Xu 2016). Large secondary repositories, such as UNITE, ISHAM DNA barcoding, and NCBI RefSeq (Targeted Loci) (Kõljalg et al. 2013, 2019; Schoch et al. 2014; Irinyi et al. 2015, 2016; O'Leary et al. 2016) became major resources for curated fungal ITS reference sequences. A major advantage of such curated databases is that curation, annotation and expansion of the database is being performed by the research community (Abarenkov et al. 2010; Irinyi et al. 2015; Nilsson et al. 2019). The ITS oligonucleotide hallmark approach attempted to refine DNA barcoding and its use in formalized interactive identification tools, by using a combination of short, species-specific sequence patterns (motifs, anchors) rather than overall sequence similarity (Druzhinina et al. 2005). This approach should be revisited as an integrated tool as it allows adjustment to situations where more than one DNA barcode is needed, and for genome-wide studies through which diagnostic short sequences may subsequently be identified.

### Lack of resolution of the ITS and use of secondary barcodes

A growing number of studies is challenging the utility of ITS for delimiting, recognizing and identifying fungal species in certain lineages (O'Donnell and Cigelnik 1997; Nilsson et al. 2008; Bellemain et al. 2010; Pino-Bodas et al. 2013; Kijpornyongpan and Aime 2016; Thiery et al. 2016; Hughes et al. 2018; Kruse et al. 2018a, b; Parks et al. 2019; Tremble et al. 2019; Stadler et al. 2020). A minor problem is that ITS may not amplify in all fungi (Kijpornyongpan and Aime 2016), but sequencing success is better than with many other markers (Schoch et al. 2012). More important caveats include lack of resolution and the potential presence of non-homologous ITS copies in the genome.

It has been demonstrated that ITS does not provide sufficient resolution among closely related species of indoor and food-borne molds (e.g. *Aspergillus*, *Penicillium*), plant or human/animal pathogens (*Alternaria*, *Cladosporium*, *Colletotrichum*, *Fusarium*, as well as *Phytophthora* in the *Oomycota*) or other fungi (e.g. freshwater *Sordariomycetes*, *Trichoderma*) including slime molds. For these, secondary barcoding markers, such as the intergenic spacer (IGS), β-tubulin II (*TUB2*), DNA-directed RNA polymerase II largest (*RPB1*) and second largest (*RPB2*) subunits, translational elongation factor 1α (*TEF1*), DNA topoisomerase I (*TOP1*), phosphoglycerate kinase (*PGK*), and cytochrome c oxidase subunit I (*COX1*) and subunit II (*COX2*), have been proposed (Table 1; Geiser et al. 2007; Gilmore et al. 2009; Damm et al. 2012; Maharachchikumbura et al. 2012; López-Quintero et al. 2013; Balasundaram et al. 2015; Choi et al. 2015; Stielow et al. 2015; Xu 2016; Al-Hatmi et al. 2016; Irinyi et al. 2016; Větrovský et al. 2016; Woudenberg et al. 2017; Schnittler et al. 2017; Tekpinar and Kalmer 2019; Luo et al. 2019; Meyer et al. 2019). Occasional cases in fungal groups where ITS otherwise provides sufficient resolution, such as the subcosmopolitan and threatened macrolichens, *Sticta fuliginosa* and *S. limbata* (Magain and Sérusiaux 2015; Moncada et al. 2020), indicate that this problem is not necessarily taxon-specific, but may denote recently or dynamically evolving lineages, which can occur in any group of fungi but is apparently more prevalent in some than in others. In recently analyzed barcode datasets (Vu et al. 2016, 2019), between 6 and 17% of yeast and filamentous fungal species were shown to be indistinguishable by ITS. Meyer et al. (2019) found that 25% of human/animal pathogenic fungi cannot be identified based on ITS alone. Many plant-parasitic lineages in *Dothideomycetes* and *Sordariomycetes* cannot be resolved to species level using ITS (Damm et al. 2012; Maharachchikumbura et al. 2012; Hyde et al. 2013; Manamgoda et al. 2014; Woudenberg et al. 2017; Haridas et al. 2020). On the other hand, for lichen-formers in *Dothideomycetes*, such as the genus *Strigula*, ITS provides a high level of resolution (Jiang et al. 2016, 2017a, b, 2020; Ford et al. 2019; Woo et al. 2020). A possible correlation between intragenomic variability of ITS and fungal life strategies should be explored further; the observed patterns indicate that fungal lineages exhibiting life strategies such as highly specific parasitism may undergo fast and complex speciation not immediately reflected in the ITS. On the other hand, economically and medically important fungi are also more densely sampled, allowing for a more fine-grained taxonomy reflecting minor but important differences between individual strains.

**Table 1:** DNA Barcoding markers proposed for fungi, their recommended nomenclature and selected examples (see also Stielow et al. 2015; Xu 2016)

DNA barcoding marker	Acronym	Examples	References
Internal transcribed spacer	ITS	universal, *Agaricus*, *Auricularia*, *Cora*, *Fomitopsis*, *Rhizoplaca*, *Sticta*	Schoch et al. 2012; Leavitt et al. 2013; Lücking et al. 2014, 2017; Moncada et al. 2014, 2020; Irinyi et al. 2016; Badotti et al. 2017
Intergenic spacer	IGS	*Schizophyllum*	James et al. 2001
β-tubulin II	*TUB2*	*Amanita*, *Aspergillus*, *Pseudopestalotiopsis*	Geml et al. 2006; Geiser et al. 2007; Maharachchikumbura et al. 2012, 2014; Fidler et al. 2017
DNA-directed RNA polymerase II subunit A	*RPB1*	*Inocybe*	Matheny 2005
DNA-directed RNA polymerase II subunit B	*RPB2*	universal, *Sordariomycetes*, *Cladonia*, *Inocybe*	Matheny 2005; Pino-Bodas et al. 2013; Větrovský et al. 2016; Luo et al. 2019
Translation elongation factor 1 alpha	*TEF1*	universal, *Sordariomycetes*, *Cantharellus*, *Fusarium*, *Trichoderma*, *Mycetozoa*	Buyck and Hofstetter 2011; O'Donnell et al. 2015; Stielow et al. 2015; Schnittler et al. 2017; Luo et al. 2019
hypothetical protein	*LNS2*	*Pucciniomycota*	Stielow et al. 2015
Phosphoglycerate kinase	*PGK*	*Fusarium*, *Penicillium*	Al-Hatmi et al. 2016; Stielow et al. 2015
DNA topoisomerase I	*TOP1*	*Pucciniomycota*, *Fusarium*, *Penicillium*	Al-Hatmi et al. 2016; Stielow et al. 2015
Cytochrome c oxidase subunit I	*COX1*	*Cladonia*, *Oomycota*, *Mycetozoa*	Pino-Bodas et al. 2013; Choi et al. 2015; Schnittler et al. 2017
Cytochrome c oxidase subunit II	*COX2*	*Oomycota*	Choi et al. 2015

In certain cases, differential levels of resolution between ITS and more variable markers is being resolved by recognizing infraspecific taxa, such as in the lichen-forming ascomycete *Thamnolia* (Onuţ-Brännström et al. 2017; Ioana et al. 2018; Jørgensen 2019); in other cases, e.g. the various IGS-defined clades of the ubiquitous basidiomycete *Schizophyllum commune* (James et al. 2001), no formal taxonomy has been implemented. As a result, the same underlying phylogenetic structure may translate into different taxonomic solutions, usually depending on the need. The level of precision to be achieved by DNA barcoding should therefore be dictated through context, regardless of how that precision is taxonomically formalized. In several fungal groups, ITS can only provide an initial approximation within a given clade, usually to a species complex, but cannot discriminate to the level of species. Two-marker barcoding systems, such as nuLSU/ITS and *TEF1* for yeasts or human/animal pathogens, are a practicable solution in such cases (Kurtzman 2006; Robert et al. 2011; Stielow et al. 2015; Vu et al. 2016; Hoang et al. 2019), although the application of this approach in metabarcoding remains challenging.

### Intragenomic variation in the ITS

More troubling than insufficient resolution is evidence of intragenomic variation of the ribosomal DNA (rDNA) cistron, including the ITS region, particularly when producing non-homologous discrete ITS variants, as this may result in conflicting molecular identifications. Intragenomic ITS variation is well-documented for bacteria, plants and animals (e.g. Wörheide et al. 2004; Rosselló et al. 2006; Stewart and Cavanaugh 2007). There is also growing evidence in certain fungal lineages (Smith et al. 2007; Simon and Weiß 2008; Lindner and Banik 2011; Kiss 2012; Vydryakova et al. 2012; Wilson et al. 2012; Harrington et al. 2014; Li et al. 2013, 2017; Kijpornyongpan and Aime 2016; McTaggart and Aime 2018; Colabella et al. 2018; Heeger et al. 2018; Hughes et al. 2018; Stadler et al. 2020). In most fungi, however, the rDNA cistron, including the ITS, appears to follow the principle of concerted evolution (Ganley and Kobayashi 2007).

Intragenomic ITS variation may largely stem from three processes: (1) stochastic point mutations resulting from DNA replication errors during cell division, (2) recombination through hybridization and introgression (e.g., McTaggart and Aime 2018), and (3) gene duplication leading to paralogs and pseudogenes (Dufayard et al. 2005). Paralogs and pseudogenes have been demonstrated for ITS, particularly in plants (Álvarez and Wendel 2003; Zheng et al. 2008; Xu et al. 2017), but convincing evidence in fungi is rare (Li et al. 2017). The distinction between hybridization and introgression or gene duplication as causes for intragenomic ITS variation is crucial, as the first may result in erroneous identifications of actually existing taxa present in an alien genome, whereas the second will produce “ghost” taxa, particularly in metabarcoding data.

Neither hybridization and introgression nor gene duplication are unique to the ITS, but the specific challenge of utilizing ITS is its presence in multiple copies in the genome, as part of 18S-ITS-28S tandem repeats located on several chromosomes. Intragenomic variation in point mutations is an obligate consequence of this, because DNA polymerases introduce stochastic errors during DNA replication. Under laboratory conditions, error rates of Taq polymerase vary between 0.1% and less than 0.01% (Chen et al. 1991; McInerney et al. 2014; Potapov and Ong 2017). With an average number of 100 copies in the fungal genome (Lofgren et al. 2019) and an average length of 550 bases (Schoch et al. 2014; Nilsson et al. 2015), the average number of bases in the entire ITS array is 55,000, so per replication cycle, 0.5 errors per ITS copy may be introduced on average. Such variation should not result in problems in ITS barcoding approaches, as it is substantially below even narrow identity thresholds. In contrast, processes such as hybridization and introgression or gene duplication introduce discrete ITS variants into the genome, which will result in serious identification errors if not properly recognized.

Intragenomic ITS variation is commonly misinterpreted, and its correct understanding is crucial for assessing potential problems. For instance, in the smut fungus *Ceraceosorus* (Kijpornyongpan and Aime 2016), intragenomic variation was found to be both stochastic and phylogenetically structured, affecting 25 and 15 out of 856 sites, respectively. Stochastic variation is a result of DNA replication errors but it does not affect phylogenetic placement of individual haplotypes when analyzed in a phylogenetic context (Lücking et al. 2014). While in the above study, the total number of stochastically varying sites (25) was high, individual sequences varied in up to four sites only, resulting in pairwise similarity of over 99.5%, thus uncritical for barcoding approaches. The 15 sites with phylogenetically structured variation resulted in the formation of three clades (Kijpornyongpan and Aime 2016). While these distinctive clades appear to represent non-homologous, discrete ITS copies, they may also be highly specific for this taxon and hence could be used for identification purpures.

Another factor concerning the impact of intragenomic variation in the ITS is the sequencing technique. In genomes dominated by one functional copy, Sanger sequencing will mask variation in spurious background signal and provide clean sequences. If several frequent haplotypes with point mutations exist, variants may appear as ambiguous base calls in specific positions with Sanger sequencing. On the other hand, discrete variants originating from hybridization or gene duplication will produce largely unresolved sequence chromatograms, requiring cloning or other techniques. In contrast to Sanger sequencing, correct interpretation of ITS variants is particularly critical in environmental metabarcoding, with the additional challenge of separating true intragenomic variation from sequencing errors (Lücking et al. 2014; Heeger et al. 2018; Thines et al. 2018). In metabarcoding approaches, natural and artifactual variants will skew diversity estimates and introduce “ghost” taxa if not properly assessed (see below). One example is the nectar yeasts (*Metschnikowiaceae*), which display high intragenomic rDNA variation (Heeger et al. 2018; Sipiczki et al. 2018), so species richness revealed through ITS metabarcoding (Vannette and Fukami 2017) will be overestimated, influencing conclusions about alpha- and beta-diversity. Similar considerations apply to other groups, such as arbuscular mycorrhizal fungi (Lekberg et al. 2014, 2018; Thiery et al. 2016). Therefore, metabarcoding data have to be interpreted with great care and multiple alignment-based approaches should be employed to identify and resolve potential issues (see below).

The availability of well-documented reference data is of particular importance to properly assess ITS variants stemming from intragenomic variation. If ITS pseudogenes have been identified for a fungal lineage (e.g. Li et al. 2017), their deposition and proper annotation will assist automated pipelines to identify such cases. Alternatively, long-fragment reads, including flanking regions of the small and/or large subunit (nuSSU, nuLSU), have been proposed as a possible solution to assess intragenomic ITS variation in metabarcoding approaches (Krüger et al. 2012; Heeger et al. 2018; Tedersoo et al. 2018b). PacBio RS produces read lengths of 3000–6000 bases, which is not sufficient to resolve intragenomic rDNA variation, as only single tandem repeats are covered, but PacBio RS II can achieve up to 60,000 bases (Rhoads and Au 2015). Given that the average number of ITS copies in the fungal genome is around 100 (Lofgren et al. 2019), PacBio Sequel II is particularly promising, as it can achieve read lengths of up to 250,000 bases, matching those obtained with Oxford Nanopore Technologies sequencing (Jain et al. 2016; Payne et al. 2019; De Coster et al. 2020; Stadler et al. 2020). While it is unclear whether the necessary high-molecular weight DNA can be obtained, since commonly used extraction techniques require a mechanical disruption of fungal cells, successful rDNA tandem repeat sequencing using a combination of PacBio and Oxford Nanopore sequencing has been performed in fungi (Wurzbacher et al. 2019). Long-fragment reads have the added advantage that nuSSU and/or nuLSU flanking regions help to anchor the ITS within a more conserved backbone (Heeger et al. 2018; Tedersoo et al. 2018b).

Another caveat of the ITS is interspecific and intragenomic length heterogeneity. In some groups, such as ascomycetous yeasts, the full length (ITS1, 5.8S and ITS2) may vary from less than 400 (*Yarrowia lipolytica*) to over 1000 bases (*Schizosaccharomyces pombe*; Esteve-Zarzoso et al. 1999). In most fungi, the length of the ITS is more uniform, but even minor variation may result in regions with low alignment confidence. Environmental metabarcoding approaches often target spacer regions only, either ITS1 or ITS2, and so short but full-length ITS reads may be unintentionally excluded from subsequent analysis by bioinformatic pipelines that by default exclude reads less than 150–200 bp long (Majaneva et al. 2015; Sinha et al. 2017; Anslan et al. 2018). Strategies to avoid this would be primer-based filtering or, as outlined above, anchoring with nuSSU or nuLSU flanking regions *via* long-fragment reads. While single-copy protein-coding markers proposed as secondary DNA barcodes in fungi do not exhibit the problems associated with multiple copies, phenomena such as paralogs may apply to them as well, such as in *COX1*, *RPB2*, and *TUB2* (Gilmore et al. 2009; Zhao et al. 2014), and their accurate interpretation likewise depends on proper data analysis and completeness of reference databases.

Regardless of the marker, the quality of reference data is of utmost importance, particularly in environmental metabarcoding. While it may not work for all fungi at the desired level of precision, ITS remains the first choice for fungal identifications at a broad level. It is not only easily amplified (with some exceptions; e.g. Kijpornyongpan and Aime 2016), but it also is the most frequently sequenced fungal marker both in specimen-based and metabarcoding approaches, making it unchallenged as a reference compared to any other marker. Even if secondary barcode markers are increasingly employed, they only represent a small fraction of available sequence data compared to ITS. GenBank currently has about 110,000 records for fungal *TEF1* and 67,000 for fungal *RPB2*, but over 1.3 million for fungal ITS. The application of ITS is thus comparable to a first diagnosis across all fungi. Depending on the results, secondary DNA barcodes may be required to obtain the desired resolution. Unfortunately, in some common and diverse fungal genera, such as *Fusarium* and *Trichoderma*, due to lack of resolution, some taxonomists have stopped sequencing the ITS. This practice is not recommended, as it excludes these taxa from being detected in metabarcoding surveys. Even if not necessarily providing enough resolution, ITS should be sequenced for each fungal lineage in addition to other markers, in order to provide a broad reference database that offers a compromise between coverage and precision. Metabarcoding studies would then employ ITS as default marker and additionally one or several secondary barcodes (e.g. Větrovský et al. 2016; Cobo-Díaz et al. 2019).

## PAIRWISE SIMILARITY ASSESSMENTS: LIMITATIONS AND SOLUTIONS

### OTU clustering

The single major issue of DNA barcoding is the routine application of pairwise similarity assessments, either through BLAST searches or clustering algorithms such as in USEARCH, VSEARCH or MultiLevel Clustering (Edgar 2010, 2013; Vu et al. 2014; Rognes et al. 2016). These approaches have become popular as they are easily integrated into automated pipelines and allow the analysis of extremely large data sets in a short time and with little manual work involved (Majaneva et al. 2015; Sinha et al. 2017; Anslan et al. 2018). In contrast to multiple alignment-based phylogenetic approaches, pairwise similarity may wrongly assess positional variation and hence not accurately reflect taxonomic entities or phylogenetic relationships. For instance, a position with a varying indel comprising either [AG], [A] or [G], in a multiple alignment will align all [A] with either [A] or a gap, but not with [G], whereas pairwise alignment will interpret a single [A] and [G] as a substitution. This issue may appear minor but can cause dramatic effects in OTU clustering, especially when such variation is caused by sequencing errors (e.g. Lücking et al. 2014). As a consequence, OTUs derived from clustering are different in number and composition when compared to actual phylogenetic entities (Porter and Golding 2011; Powell et al. 2011; Lücking et al. 2014). Huse et al. (2010) designed a two-step clustering approach that reduces the effect of OTU inflation in de-novo clustering. Swarm (Mahé et al. 2014, 2015) reduces the issue of random effects on cluster formation and inflation. Increased accuracy while not compromising in computational speed can also be achieved by hc-OTU clustering through homopolymer compaction (Park et al. 2016). Employing PaPaRa (Berger and Stamatakis 2012; Wegmann 2019) in read processing can substantially reduce sequencing errors prior to OTU clustering: Lücking et al. (2014) found that after automated removal of homopolymer-based errors using PaPaRa, OTU clustering accuracy improved by 94%. Post-processing of clusters to filter out potentially artifactual OTUs can be performed with the LULU package (Frøslev et al. 2017).

Clustering approaches require predefined similarity thresholds, but such fixed thresholds do not exist when it comes to the delimitation of species. In phylogenetic treatments based on ITS, sister species can differ in as few as three bases (around 99.5% similarity; Garnica et al. 2016; Lücking et al. 2017; Urbina and Aime 2018; Vu et al. 2016, 2019). Indeed, in certain groups of fungi, such as *Hypocreales* (*Fusarium*, *Gibberella*, *Trichoderma*), species hypotheses delimited at 98.5% in UNITE include sequences from type material of several to numerous different species (Robbertse et al. 2017). Varying optimal thresholds have been determined for different lineages based on two large barcode datasets (Vu et al. 2016, 2019). If the marker of choice lacks resolution, then even the highest similarity threshold will not yield reliable OTU estimates. Clustering approaches set the threshold at either 97%, the default in most pipelines (Majaneva et al. 2015; Sinha et al. 2017; Anslan et al. 2018), or at 98.5%, the default used in curated databases, such as UNITE and ISHAM DNA barcoding for “species hypotheses” based on ITS (Kõljalg et al. 2013, 2019; Irinyi et al. 2015; Jeewon and Hyde 2016). This latter threshold does reflect empirically derived estimates (e.g. Lücking et al. 2020; and Fig. 3); the aforementioned analysis of 9000 yeast cultures showed that a threshold of 98.41% similarity (towards the corresponding type strain) for the ITS worked well for most species (Vu et al. 2016).

The potential underestimation of species richness using fixed pairwise similarity thresholds is counterbalanced by the overestimation of taxonomic units through OTU clustering bias. As a result, a proportion of OTUs may not be real taxonomic entities, whereas a proportion of real taxonomic entities may be missed. This situation is further complicated in lineages characterized by high heterogeneity of ITS sequences (sometimes more than 10%; Thiery et al. 2016; Sipiczki et al. 2018). Arbitrary variation of predefined thresholds, e.g. between 97 and 98.5%, will further affect the recovery of taxonomic entities in clustering approaches (Lücking et al. 2014; Garnica et al. 2016; Edgar 2018).

### BLAST mapping

Similarity assessment through pairwise alignment also poses limitations for BLAST-based identifications of individual amplicon variant metabarcoding reads (Callahan et al. 2017), such as implemented in BLAST+, the RDP Bayesian classifier or *MycoBank* BioloMICS Sequences (Camacho et al. 2009; Robert et al. 2013; Deshpande et al. 2016). While amplicon variant BLAST mapping avoids potential bias of OTU clustering, it also relies on pairwise alignment scores, particularly max score, query cover, e value and percentage identity. Max score, the sum of match rewards and mismatch and gap penalties, depends on query and reference sequence length: shorter matches with higher identity may receive a lower score and not be immediately visible as best hits. The e value, the number of expected hits of similar score that could be found by chance, is computed from max score and results in the same sorting of matches but depends on query sequence length and reference database size and hence is not comparable across databases. Both max score and e value are also affected by the structure of reference sequences, such as partial ITS sequences that include long portions of the conserved nuSSU or nuLSU or are dominated by the 5.8S region. Algorithms that extract the diagnostic ITS spacer regions, such as the FungalITSextractor (Nilsson et al. 2010) and ITSx (Bengtsson-Palme et al. 2013), address this issue: metabarcoding pipelines that contain FungalITSextractor (Bálint et al. 2014) or ITSx (Hildebrand et al. 2014; Gweon et al. 2015; Anslan et al. 2017) perform best in relation to BLAST mapping (Anslan et al. 2018).

Percentage identity can be measured in three ways: (1) N_matches_ / N_total pairwise alignment length_ (BLAST identity), (1) N_matches_ / N_total pairwise alignment length minus indels_ (gap-excluded identity), and (3) N_matches_ / N_total pairwise alignment length minus indel groups_ (gap-compressed identity). BLAST identity considers individual indels as mismatches and hence results in lower similarity values than the other two approaches for a given sequence pair. It is also more sensitive to homopolymer-based sequencing errors in the query reads and affected by improper trimming of low-quality terminal portions of reference sequences (Nilsson et al. 2017). As a result, sequences retrieved as best hits in BLAST searches are not necessarily most closely related (e.g. Thiery et al. 2016; Lücking et al. 2020). The above issues also depend on whether query and reference sequences represent the full ITS or only the ITS1 or ITS2 spacer regions (Nilsson et al. 2008; Blaalid et al. 2013; Tedersoo et al. 2015; Garnica et al. 2016; Badotti et al. 2017; Větrovský et al. 2020).

Even so, BLAST is the most commonly employed read mapping technique, either against a primary sequence repository, such as GenBank or against curated or otherwise specialized databases, such as UNITE. Notably, reported problems can largely be solved by increasing the quality and representativity of reference databases, in particular correct sequence labeling, and by adding a verification step (Lücking et al. 2020). The latter is not possible for metabarcoding studies, as BLAST results cannot be inspected individually. However, automated verification can be achieved through phylogeny-based analysis of metabarcoding reads that compute statistical support values for alternative placements. This can be achieved either through local alignments of BLAST hits under a Bayesian framework (Munch et al. 2008; Porter and Golding 2011), with a probabilistic approach such as PROTAX Fungi (Abarenkov et al. 2018), through a "random forest" learning tool (Meher et al. 2019), or through read placement into a separately established reference tree (Berger et al. 2011; Matsen et al. 2012; Barbera et al. 2019).

### Multiple alignment-based read placement

Read placement into a reference tree is a promising approach that increases accuracy and precision in metabarcoding studies compared to OTU clustering and BLAST-based amplicon variant read mapping (Stark et al. 2010; Berger et al. 2011; Matsen et al. 2012; Paul et al. 2018; Czech et al. 2018, 2019; Barbera et al. 2019; Carbone et al. 2019). The method, also dubbed phylogenetic binning, relies on three components: (1) a reference tree for a set of taxa which can be derived through phylogenetic analysis of existing data; (2) a fixed alignment of reference sequences corresponding to the metabarcoding marker (e.g. ITS) for the taxa included in the reference tree; (3) a set of query reads from a metabarcoding study corresponding to the same barcoding marker. In a first step, the query reads are automatically aligned to the fixed reference alignment (Berger et al. 2011), using for instance PaPaRa (Berger and Stamatakis 2012) and the [−-add] function in MAFFT (Katoh and Frith 2012). In a second step, each query sequence is individually placed into the reference tree based on its alignment by invoking the *Evolutionary Placement Algorithm* (EPA; Stamatakis et al. 2010; Berger et al. 2011; Barbera et al. 2019). In addition to a maximum likelihood or maximum parsimony approach offered by the EPA, read placement can also be performed in a Bayesian framework using *pplacer* (Matsen et al. 2010). Mirarab et al. (2012) proposed SATé-enabled phylogenetic placement (SEPP) to improve alignment accuracy through simultaneous alignment and tree building.

Phylogenetic binning placed each query sequence at the most closely matching node under an evolutionary model: if the query sequence matches a terminal, it will cluster with that terminal; alternatively, it attaches to an internal node representing a higher taxonomic level, an approach that conceptually corresponds to the LCA. While the Bayesian framework in *pplacer* offers direct assessment of statistical confidence, the EPA allows the computing of bootstrap support values for potential alternative read placements. These options provide an automated, quantitative verification step not available through OTU clustering or BLAST mapping, except with approaches such as PROTAX Fungi and “random forest” learning (Abarenkov et al. 2018; Meher et al. 2019). Optionally, prior to invoking the EPA, the phylogenetic pattern of the metabarcoding marker over the fixed reference alignment can be analyzed using a maximum parsimony or maximum likelihood approach in order to compute a weight vector. In doing so, potential homoplasy through saturation in highly variable regions of the metabarcoding marker can be assessed to improve the subsequent placement of query sequences into the reference tree. Therefore, the reference tree should be inferred based on markers that do not include the metabarcoding marker, to avoid circular conclusions.

Apart from bootstrapping and Bayesian posterior probabilities offering automated verification, phylogenetic binning has further, important advantages over OTU clustering and BLAST mapping. Point variation in query reads, whether representing sequencing errors or real variation, does not prevent their accurate placement into a reference tree (Berger et al. 2011; Lücking et al. 2014). The absence of close relatives in a reference tree is immediately discernible by placement of a query read at a deeper node, a more accurate approach than LCA, as it avoids the ambiguity of low similarity values in the latter. Read placement also allows the implementation of quantitative species delimitation methods to automatically assess taxonomic diversity, an approach already integrated into the phylogenetic binning approach (Zhang et al. 2013). Broad reference trees can be assembled and centrally maintained to be used in analytical pipelines (Tedersoo et al. 2018a; Carbone et al. 2019), or alternatively computed automatically from published sequences (Czech et al. 2019), allowing dynamic on-the-fly solutions for particular situations.

Given the large amount of data to be analyzed, often encompassing hundreds of thousands of reads, environmental metabarcoding of fungi requires a trade-off between speed on one hand and accuracy and precision on the other (see below). Up to the recent past, OTU clustering was the only viable approach to achieve this goal. However, phylogenetic binning is now possible through massive parallel computing on large clusters (Barbera et al. 2019; Carbone et al. 2019) and may become the method of choice for metabarcoding studies. Even when OTU clustering and/or BLAST mapping are preferred, certain strategies can help to improve results, including PaPaRa read processing to remove specific sequencing errors, algorithms such as FungalITSextractor and ITSx to increase diagnostic power, taxon-specific dynamic pairwise similarity thresholds, the analysis of a given sample with both the ITS and secondary barcodes, and locally aligning and analysing BLAST hits using automated phylogenetic approaches.

## CONCLUSIONS AND RECOMMENDATIONS

As is true for other organisms, fungal species are not only defined horizontally through phylogenetic and phenotypic coherence, but also vertically through time of origin and subsequent diversification. Individually different evolutionary histories thus make it impossible to apply universal and unambiguous criteria for the delimitation, recognition, and identification of fungi. Best practice depends on each group, and residual ambiguity remains in many cases, also due to incompleteness of identification tools and reference data. The desire for rapid, automated approaches, such as OTU clustering and pairwise similarity-based BLAST mapping amplifies these problems.

Full exploration of the various conceptual approaches to delimit fungal species, including reproductive biology, is currently only feasible for selected taxa including model organisms. Since generalizations from model studies are limited to close relatives or ecologically equivalent taxa, this approach should be expanded to cover selected species in all groups of fungi, representing the diversity of phenotypes, lineages, and nutritional strategies. For broadly cataloguing fungal diversity, an integrative (polyphasic) taxonomic approach seems most effective, adjusted to the group under study and combining molecular and phenotype data. In many groups, single-marker DNA barcoding may suffice, whereas more complex taxa require a combination of primary and secondary barcoding markers or multi-marker approaches. Phylogenomics may be employed to resolve particularly difficult species complexes, but this approach demands large computational and personal resources and is currently limited to exemplar case studies.

The phenotype remains an integrative component of fungal taxonomy, encompassing also data derived from cultures and other sources. Taxonomists will continue to describe new species in the absence of molecular data, in groups where this approach is justified. However, phenotypic data should be thoroughly analyzed before establishing new species by any method. If the material would allow the generation of molecular data but the methodology to do so is not available, then collaboration to produce such data is recommended. In general, the goal remains to document all fungi with molecular data. Phenotypic data are of particular importance when assessing the status of phylogenetically distinct clades through integrative taxonomy. In such cases, quantitative analysis of structured phenotype matrices should be implemented to assess phenotypic variation in a phylogenetic context, which will then also allow the detection of reliable diagnostic characters.

On a molecular level, ITS remains the universal fungal barcode marker to initially identify phylogenetic lineages. It can thus be considered a first diagnosis. Where ITS does not suffice to discriminate between species, secondary barcoding markers or multi-locus approaches need to be employed to achieve the desired level of precision and accuracy. How individual markers resolve species is determined by context, and feasibility of particular markers should not be uncritically transferred from one taxonomic group to another but instead empirically explored for each taxon. ITS will likely remain the marker of choice for fungal metabarcoding studies, although long-read approaches or the addition of secondary barcoding markers will improve accuracy and precision. However, metabarcoding approaches should move away from OTU clustering and BLAST mapping exercises and instead implement phylogenetic methods, such as read placement (phylogenetic binning).

Current issues arising with DNA barcoding of fungi are not primarily due to conceptual limitations of the approach but due to shortcomings of reference databases, including incompleteness in terms of taxonomic coverage, lack of properly documented genetic diversity, and inaccuracy of sequence labels. Major efforts must therefore be directed at further improving these resources, particularly the continued and critical revision of existing data to achieve high quality labels.

## Data Availability

Data sharing is not applicable to this article as no datasets were generated or analysed specifically for this purpose.
